# Arsenic Neurotoxicity in Humans

**DOI:** 10.3390/ijms20143418

**Published:** 2019-07-11

**Authors:** Hitoshi Mochizuki

**Affiliations:** Division of Neurology, Respirology, Endocrinology and Metabolism; Department of Internal Medicine; Faculty of Medicine; University of Miyazaki, Miyazaki 889-1692, Japan; mochizuki-h@umin.net; Tel: +81-985-85-2965; Fax: +81-985-85-1869

**Keywords:** arsenic, neurotoxicity, Toroku, mechanism, oxidative stress, metabolite pathway

## Abstract

Arsenic (As) contamination affects hundreds of millions of people globally. Although the number of patients with chronic As exposure is large, the symptoms and long-term clinical courses of the patients remain unclear. In addition to reviewing the literature on As contamination and toxicity, we provide useful clinical information on medical care for As-exposed patients. Further, As metabolite pathways, toxicity, speculated toxicity mechanisms, and clinical neurological symptoms are documented. Several mechanisms that seem to play key roles in As-induced neurotoxicity, including oxidative stress, apoptosis, thiamine deficiency, and decreased acetyl cholinesterase activity, are described. The observed neurotoxicity predominantly affects peripheral nerves in sensory fibers, with a lesser effect on motor fibers. A sural nerve biopsy showed the axonal degeneration of peripheral nerves mainly in small myelinated and unmyelinated fibers. Exposure to high concentrations of As causes severe central nervous system impairment in infants, but no or minimal impairment in adults. The exposure dose–response relationship was observed in various organs including neurological systems. The symptoms caused by heavy metal pollution (including As) are often nonspecific. Therefore, in order to recognize patients experiencing health problems caused by As, a multifaceted approach is needed, including not only clinicians, but also specialists from multiple fields.

## 1. Introduction

Arsenic (As) has a long history of use as a pigment and as a homicidal agent. However, in the past 100 years, As has been used as a pesticide, medicine, and component of a number of products [1]. As the global population increasingly relies on aquifers for drinking water, and because some aquifers are contaminated by heavy metals, the population exposed to As has increased dramatically [2]. In addition, reliance on the excavation of deep strata when mining rare metals has increased human contact with heavy metals. Volcanic eruptions can also affect heavy metal exposure, and together, these causes dramatically increase the chances of human contact with heavy metals in amounts far above acceptable thresholds for human health.

Among heavy metals, As is attracting media attention owing to its high toxicity. At least 140 million people in more than 50 countries are exposed to As-contaminated drinking water [3]. However, although a small number of acute As exposure patients have been studied in detail, relatively few studies have been performed on a detailed neuropathy of patients chronically exposed to As [4,5,6]. Although patients with chronic As exposure are numerous, their symptoms and clinical courses remain unclear. Medical care of patients with chronic As exposure is often performed through trial and error. In this review, we describe not only the research on As toxicity, but also clinical aspects and case studies with the goal to make this review useful to physicians who examine patients with arsenicosis as well as to researchers.

## 2. Arsenic in the Environment

Arsenic is widely distributed throughout the earth. In the crust, it often exists in its trivalent atomic state, inorganic As (III), together with other metals such as copper, lead, and iron. In soil and water, it is generally oxidized to pentavalent As (V). In low oxygen environments, such as deep well water or deep seawater, it is reduced to trivalent As (III). Sea water has an As concentration of approximately 2 ppb [7], whereas rain and river water have almost 0 ppb [6]. Despite these levels, the area most prescient for researchers remains As contamination of aquifers due to exposure risk.

Arsenic accumulation in animals sheds light on the significance of the different chemical species of As. Land animals contain 0.06–0.4 ppm of As, whereas fish and shellfishes contain 0.78–25 ppm [8]. Although the As quantities in fish and shellfish are much higher than that in land animals, the form in fish and shellfish is mostly organic As of arsenobetaine (C5H11AsO2). Arsenobetaine is neither metabolized by nor accumulated in humans, and thus, it is considered non-toxic to humans [9]. 

## 3. Arsenic Metabolic Pathway and Toxicity

### 3.1. Metabolic Pathway

Arsenic metabolites exist both in organic and inorganic forms, and both types can exist in either trivalent or pentavalent oxidation states. Thus, there are a variety of molecular species that have different biological effects, which further complicates diagnoses. 

**Figure 1:**
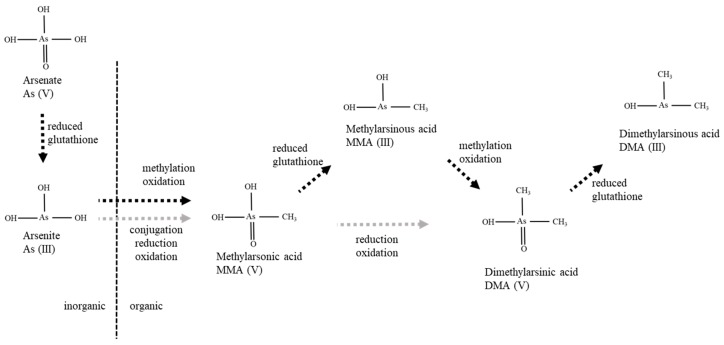
Proposed metabolic pathways for the conversion of inorganic Aresenic (As) into organic As. The mechanism involved in the oxidation and reduction of As is shown.

The exact metabolic pathways of As are yet to be confirmed in humans and food animals, although the proposed metabolic pathway of As is shown in Figure 1 [10,11]. Oxidative methylation and glutathione conjugation are believed to be the primary pathways of As metabolism [12,13]. Inorganic As (V) is known to reduce to As (III), which is a prerequisite for methylation in mammals. Inorganic As (III) is methylated to methylarsonic acid (MMA) and dimethylarsinic acid (DMA) by alternating the reduction of pentavalent As to trivalent As (Figure 1). In some species (though not in humans), DMA can be converted into trimethylarsine oxide during oxidative methylation [14]. 

In humans, the bioavailability of inorganic As is 60%–87% [15,16], and inorganic As and its metabolites are mainly excreted in urine and bile. The biological half-life of As is approximately 4 days, depending on the form: arsenite is believed to have a shorter half-life compared to arsenate [17]. The most frequently detected As compounds in human urine are DMA (V) (40%–80%), MMA (V) (10%–25%), and inorganic As (10%–30%) [18,19]. Arsenosugar and/or arsenobetaine are other concerning forms of As that people may be exposed to when eating algae or seafood—these forms are excreted in the urine.

### 3.2. Toxic LD50 Concentrations

Methylation is generally considered to be the primary detoxification pathway for inorganic As, however, the toxicity levels of inorganic and organic As metabolites are mixed. For example, several studies have demonstrated that trivalent As is more toxic than the pentavalent state [1]. Trivalent As compounds, As (III), MMA (III), and DMA (III) are thought to interact with thiol groups of proteins and enzymes and inhibit the catalytic activity of enzymes [20]. The toxicity of these metabolites were investigated in Chang human hepatocytes using a lethal dose, 50% (LD50) in the three cytotoxicity assays (LDH, K+ and XTT) [21]. The order of toxicity obtained was as follows: MMA (III) > As (III) > As (V) > MMA (V) = DMA (V). Similar findings were observed in another study in which LD50 concentrations of As (III), As (V), MMA (III), MMA (V), DMA (III), and DMA (V) were 50 µM, 180 µM, 8 µM, 60 mM, 8 µM, and 15 mM, respectively [22]. This stands in contrast to other As chemical species—arsenobetaine and arsenosugar—that were judged as non-toxic. In animal experiments, it was concluded that MMA (III) and DMA (III) are more toxic than inorganic As compounds and induce chromosomal mutations but not gene mutations [23].

The residents of Kamisu City, Japan (*n* = 157) were orally exposed to diphenylarsinic acid (DPAA; C_12_H_11_AsO_2_) via the ingestion of contaminated groundwater. Subsequently, a clinical syndrome associated with cerebellar and brainstem symptoms was observed in 20 of the 30 residents who consumed high concentrations of DPAA in the contaminated well water [24]. After this DPAA leak accident, the toxicity of organic and inorganic As were examined using human cervical carcinoma HeLa cells by the Japanese government [25]. Using a relative scale, with the toxic level of DPAA defined as “1,” the levels of As (III), As (V), MMA (V) and DMA (V) were 96, 5.8, 0.18, and 1.0, respectively. 

## 4. Toxic Mechanisms

The underlying mechanisms of As-induced neurotoxicity mostly remain unknown, though several mechanisms have been proposed, mainly from animal experiments. Metabolites exert their toxic effect by inactivating a host of enzymes, especially those involved in the cellular energy pathway as well as DNA synthesis and repair [26]. Several mechanisms—oxidative stress, thiamine deficiency, and decreased acetyl cholinesterase activity—seem to play key roles in As-induced neurotoxicity [27,28]. 

### 4.1. Mitochondrial Dysfunction

One of the most important mechanisms involved in the neurotoxicity of As is its ability to cause oxidative stress and mitochondrial dysfunction [29,30]. Arsenic decreased the activities of mitochondrial complexes I, II-III, and IV in the rat brain and increased the levels of reactive oxygen species (ROS) [31]. The accumulation of ROS is responsible for lipid bi-layer damage and it causes mitochondrial swelling and a drop in the membrane potential [32]. It has also been shown that oxidative stress and mitochondrial dysfunction may cause neurodegeneration [33].

### 4.2. Lipid Peroxidation

Oxidative stress and the resulting lipid peroxidation are involved in various pathological states including inflammation, atherosclerosis, neurodegenerative diseases, and cancer [34]. Lipid peroxidation is a basic cellular deterioration process induced by oxidative stress [35]. Lipid peroxidation induced by oxidative stress due to As exposure leads to DNA damage and subsequent brain cell death, and it induces the degeneration of the central nervous system (CNS) [36]. In addition, plasma lipid peroxidation has been shown to be positively correlated with As levels in urine [37].

### 4.3. Apoptosis

Apoptosis is a cellular response to maintain normal cell development and proper function of multicellular organisms. Arsenic neurotoxicity involves the induction of apoptosis by activating p38 mitogen-activated protein kinase and JNK3 pathways [38]. In another study using HepaRG cells, the DMA (III) exposure increased the activity of caspase-9, an apoptosis initiator caspase [39]. Exposure to As reduced rat cerebellar neuron viability and induced nuclear fragmentation and condensation as well as DNA degradation to oligonucleosome fragments, which are processes associated with apoptosis. Together, these studies indicate that As-induced apoptosis may be related to As neurotoxicity in humans.

### 4.4. Increased Calpain

Inorganic As (III) causes compositional changes in sciatic nerve proteins, such as reduction in NF-L expression [40]. Furthermore, in vitro studies with various As metabolites have shown that MMA (V) and DMA (V) affect the expression of neurofilaments and tau genes, but not inorganic As (III) [41]. In animal experiments, As exposure reduced the expression of the neurofilament protein and induced destabilization and disruption of the cytoskeletal framework which may eventually lead to the axonal degeneration of peripheral nerves [42]. It has been speculated that the cleavage of p35 is caused by calpain activation, which is induced by Ca^2+^. The inhibition of calpain by calpeptin prevents the cleavage of p35 to p25. These results suggest that cleavage of p35 to p25 by calpain, likely promotes As-induced Ca^2+^-influx, and therefore, it may be the mechanism by which As induces its neurotoxic effects [41].

### 4.5. Thiamine Deficiency

The deficiency of thiamine (vitamin B1) induces neuronal complications, and As causes thiamine deficiency and inhibits pyruvate decarboxylase [43], an enzyme responsible for converting glucose to energy. Trivalent As inhibits enzyme complexes through ROS. ROS production causes pyruvate dehydrogenase inactivation through oxidation, which can occur at a much lower concentration than arsenite binding directly to the critical thiols [44,45]. Axonal neuropathy, which is similar to beriberi neuropathy or mild Wernicke’s encephalopathy, may be induced by thiamine deficiency and the inhibition of pyruvate decarboxylase due to As exposure.

### 4.6. Decreased Acetylcholinesterase Activity

Acetylcholinesterase is one of the many important enzymes needed for the proper functioning of the human nervous system. In rats, As trioxide significantly decreased the activity of serum acetylcholinesterase in a dose-dependent manner [46]. The decreased acetylcholinesterase activity caused cholinergic crisis, which may be associated with peripheral neuropathy or CNS damage [28,46]. There are several possible mechanisms of toxicity, and the correspondence between the mechanisms and the symptoms remains unclear. 

## 5. Clinical Neurological Symptoms

**Figure 2:**
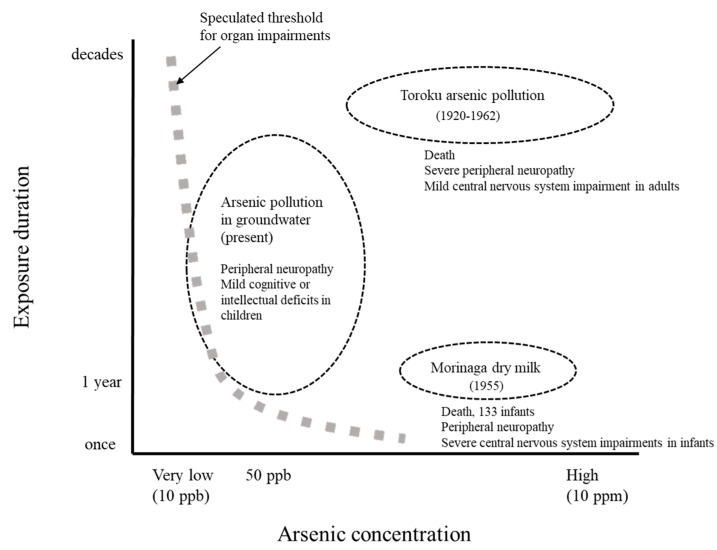
Duration and concentration of As pollution and As exposure incidents. Speculated thresholds for organ impairments are shown as a thick gray dotted line.

Peripheral neuropathy due to chronic As exposure is caused by drinking water with As concentrations as low as 10–50 ppb [6]. The resulting impairment is observed predominantly in sensory fibers, and less so in motor fibers [5,47]. Sural nerve biopsies revealed a reduction in both small myelinated and unmyelinated fibers, which occurred with the axonal degeneration of peripheral nerves [47,48]. CNS impairment may occur at 50 ppb or more in children [49], though in adults, CNS impairments are only known to be caused by As exposure at high concentrations [50]. Peripheral neuropathy due to As exposure may recover in the long term, however, CNS impairments are less likely to recover. Organ damage is related not only to As exposure concentrations, but also to acute or chronic factors (Figure 2).

### 5.1. Acute As Poisoning

Oral exposure to As is associated with gastrointestinal symptoms including cramps, nausea, vomiting, and diarrhea and with cardiovascular and respiratory symptoms such as hypotension, shock, pulmonary edema, and heart failure [51]. In acute As poisoning, death is usually due to cardiovascular collapse and hypovolemic shock. The fatal human dose for ingested As trioxide is 70–300 mg [18,26]. After the ingestion of a lethal dose, death occurs after 12–24 h. Acute As exposure also includes neurological symptoms such as light-headedness, delirium, encephalopathy, muscle weakness or cramping, and peripheral neuropathy [52]. Peripheral neuropathy occurs as symmetrical sensory-motor polyneuropathy one or more weeks after the initial toxic exposure, which usually shows axonal degeneration but sometimes shows demyelinating polyradiculoneuropathy-like Guillain–Barré syndrome [53].

### 5.2. Toroku As Pollution

Toroku is a small village in a narrow valley in Miyazaki prefecture, Japan with a total population of less than 300. Arsenic was mined intermittently and refined at the Toroku mine between 1920 and 1941 and between 1955 and 1962. The roasters used at the mine’s refinery were primitive and lacked dust-collecting systems. Therefore, residents were exposed to very high concentration of As via air, food, water, and skin contact. Dozens of people died at a young age, mainly the workers and residents near the mine. Although As concentrations in the environment were not measured until 1962, they were investigated by Miyazaki prefecture in 1972 [54]. The average As concentrations in the neighboring soil and in the water percolating from the slag were 2,760 mg/kg and 180 mg/L, respectively. 

Since 1974, Miyazaki prefecture has been conducting medical examinations for residents in the district, and according to the data, subjective symptoms such as sensory disturbances, skin lesions, upper airway symptoms, hearing impairments and dizziness have been present in over 85% of chronically exposed patients [5]. In terms of sensory impairments, only 30% of the patients were judged to be objectively abnormal by neurological examination. Studies using somatosensory-evoked potentials showed that the prolongation of the central sensory conduction time, which indicates sequelae in the CNS, may remain even after more than 40 years post-As exposure [50]. Similarly, more than 40 years after the final As exposure, 50% of the residents had hearing impairment, however, no significant differences were observed in auditory brainstem response from the normal group [55]. In the determination of sequelae in elderly patients, it is difficult to distinguish them from typical age-related phenomena.

### 5.3. Arsenic Poisoning in Morinaga Dry Milk

In the early summer of 1955, physicians in the western part of Japan became worried about outbreaks of an unusual disease characterized by anorexia, skin pigmentation, diarrhea, vomiting, fever, and abdominal distention among infants, most less than 12 months of age [56]. It was determined that Arsenic (V) was inadvertently added to powdered milk products made by the Tokushima plant of the Morinaga Milk Industry. The company used an alternative low-cost industrial dibasic sodium phosphate as a stabilizer which was added to the infant powdered milk products. It was found that As was also used as a catalyst in the manufacturing process. The As concentration in the milk was 4–7 mg/L (4000–7000 ppb) [56]. The As intake for the exposed infants was estimated to be 1.3–3.6 mg/day, and the total intake was estimated to be 90–140 mg. In a long-term follow-up study, skin disorders such as keratosis, as well as central nervous disorders such as deafness, mild brain damage, mental retardation, and epilepsy remained [56,57]. Generally speaking, neurological impairment induced by As has been reported as peripheral neuropathy [4,5,58]. However, in the Morinaga milk incident, severe CNS impairments were induced, likely due to the very high concentration of As and the immature blood-brain-barrier of the infants.

### 5.4. Arsenic Contamination in Groundwater 

Unfortunately, As contamination in groundwater is now a common phenomenon being reported from various countries, including Bangladesh, India, Myanmar, Argentina, Chile, China, Hungary, Mexico, Nepal, Taiwan, the United States, and others. At least 140 million people from 50 countries are exposed to As through low-dose As-contaminated groundwater at levels above 10 ppb [3]. Several studies have shown that As exposure induces peripheral neuropathy or neuritis [4,58,59,60]. The type of neuropathy caused by such extremely long exposure to low As concentrations in water has gradually become clear over the last decade. For neurological impairments, it has been suggested that mild peripheral neuropathy may occur by drinking As-contaminated water at the level of 10 ppb [6]. On the other hand, there is no study showing that CNS impairments occur due to drinking As-contaminated groundwater in adults [4,6,61] except the DPAA exposure of the Kamisu city incident [24]. In a study in Cambodia, neurobehavioral function was found to be affected in the group of children that consumed more than 50 ppb of As-contaminated drinking water compared to those in the normal control groups [49]. The long-term prognosis for the above impairments is unknown.

## 6. Exposure Dose–Response Relationship in Various Organs

Exposure dose–response relationships of As have been described in previous studies [62]. There are significant As exposure dose–response relationships for the occurrence of skin lesion, internal malignancies, vascular diseases, and elevated hepatic enzyme levels [62,63,64]. However, the comparisons of these studies are difficult because the exposure period is different among these studies, and acute and chronic As exposure have distinct clinical symptoms [26]. Furthermore, the longer the exposure period, the lower is the threshold at which organ impairments might occur (Figure 2) [26,61]. However, the damage and the mechanism of the effects of high As concentrations with short-term exposure would be different from those of low concentrations with long-term exposure. 

In the studies of chronic As exposure, an increased prevalence of skin lesions was observed in people drinking As-contaminated groundwater at a level of 5–10 ppb [62,65]. In the analysis of internal malignancies and As exposure, the dose–response relationships for the occurrence of lung, bladder, and kidney cancers were linear [62,66,67]. A threshold level for inorganic As in the drinking water for these cancers is estimated to be between 50 and 150 ppb [68]. In a survey of 1,185 people in the United States, those who consumed As-contaminated water of more than 10 ppb were statistically more likely to report a history of circulatory problems [69]. Long-term exposure to As from drinking water has been shown to have a dose–response relationship with an increased risk of diabetes, mellitus, and hypertension [70]. A significant As exposure dose–response relationship was also observed in serum hepatic enzyme levels, with statistically higher levels found in subjects who consumed As-contaminated water of more than 34 ppb [63]. In the context of neurological impairments, subjective neurological impairments occurred at As contamination levels of around 10 ppb, and objective peripheral nerve disturbances occurred at more than 50 ppb [6]. 

## 7. Effect on Children

There are no conclusions as to whether the intake of low concentration As-contaminated drinking water adversely affects the brain of children. An epidemiological study indicated that CNS impairments such as cognitive or intellectual deficits were associated with As exposure in children [71,72,73]. However, a study in West Bengal showed no association between long-term As exposure in water and intellectual functions in children [74]. 

To discuss the effect of As exposure on children, we will contrast a few differences between adults and children. First, exposure durations in children are shorter than those in adults. If toxic effects are cumulative, adults would be affected more severely than children. Second, children may have a higher As methylation capacity than adults [75,76], resulting in more efficient detoxification [76] and a lower incidence of neuropathy. In fact, in the case of the Wakayama curry-poisoning cases, the majority of the children were in the process of recovery approximately 1 week to 10 days after the high dose As-contaminated curry intake, whereas the poisoning symptoms in adults were exacerbated [77]. Third, compared with adults, children have an immature defense system of the blood–brain–barrier against toxic substances. Therefore, CNS damage due to As may occur easily in children. Therefore, when determining the reference value of drinking water, it is necessary to carefully consider whether the value for children is the same as that for adults.

## 8. Factors to Consider

When considering the effects of As on humans, the degree of injury varies depending on the route, concentration and duration of exposure, the total amount, and the target organ. Patient factors such as nutrition, age and general health status may also amplify or diminish the ill effects of As exposure [78]. The protection provided to the CNS by the blood–brain barrier is impaired if exposed to high concentrations, however, if exposed to low concentrations, damage may not occur easily, even if exposed for a long time. Among the damaged organs are those that can be expected to regenerate, such as peripheral nerves and the liver, and those that are difficult to regenerate, such as the CNS. It is also necessary to consider the effects of heavy metals other than As. At the As polluted area, there is often contamination with other toxic heavy metals such as lead, manganese, cadmium, chromium, uranium, and copper [49,54,79] which may have compounding effects on As contamination.

## 9. Conclusions

Arsenic-contaminated drinking water has long been a global problem, especially in South Asia. To evaluate the health damage caused by heavy metals in drinking water, we estimated the residents’ clinical findings based on past data. However, several factors such as exposure route, As quantity, characteristics of the patients and their organs are intricately intertwined. The emerging symptoms are often nonspecific and the diagnoses require a different public health approach than the conventional clinical approach. To determine whether health problems in certain residents or patients caused by As, a multifaceted approach is needed, including not only clinicians but also specialists from multiple fields.
